# The MetaboHealth Score Enhances Insulin Resistance Metabotyping for Targeted Fat Loss: The PERSON Study

**DOI:** 10.1002/oby.70116

**Published:** 2026-01-14

**Authors:** Jordi Morwani‐Mangnani, Fatih A. Bogaards, Alexander Umanets, Gabby B. Hul, Anouk Gijbels, Gijs H. Goossens, Joris Deelen, Marian Beekman, Lydia Afman, Ellen E. Blaak, P. Eline Slagboom

**Affiliations:** ^1^ Section of Molecular Epidemiology, Department of Biomedical Data Sciences Leiden University Medical Center Leiden the Netherlands; ^2^ Division of Human Nutrition and Health Wageningen University Wageningen the Netherlands; ^3^ Centre for Healthy Eating and Food Innovation Maastricht University‐Campus Venlo Venlo the Netherlands; ^4^ Department of Human Biology Institute of Nutrition and Translational Research in Metabolism (NUTRIM), Maastricht University Medical Center^+^ Maastricht the Netherlands; ^5^ TI Food and Nutrition (TIFN) Wageningen the Netherlands; ^6^ Max Planck Institute for Biology of Ageing Cologne Germany; ^7^ Cologne Excellence Cluster on Cellular Stress Responses in Ageing‐Associated Diseases (CECAD), University of Cologne Cologne Germany

**Keywords:** aging, body composition, DXA, insulin resistance, metabolic disease, metabolomics

## Abstract

**Objective:**

We previously identified distinct muscle and liver insulin resistance (IR) metabotypes in middle‐aged and older adults. The PERSON study showed that a low‐fat, high‐protein, high‐fiber diet benefits the muscle IR group, while a high‐monounsaturated fatty acid diet benefits the liver IR group. We also developed the MetaboHealth score, reflecting risks of mortality, frailty, and cognitive decline. This study aimed to examine whether MetaboHealth interacts with IR metabotypes to influence (i) cardiometabolic health and (ii) body composition outcomes in the PERSON study, informing precision nutrition strategies.

**Methods:**

In total, 242 adults aged 40–75 with IR were randomized to follow an isocaloric low‐fat, high‐protein, high‐fiber or high‐monounsaturated fatty acid diet for 12 weeks. Of these, 184 with complete data were grouped into MetaboHealth tertiles (higher = poorer health). Outcomes included a 7‐point oral glucose tolerance test and DXA‐based body composition. Linear mixed models assessed four‐way interactions.

**Results:**

No interaction was observed for cardiometabolic outcomes. Significant interactions were found for android, gynoid, total fat percentage, and fat mass index. In the healthiest tertile, matched diets led to greater fat loss. In the poorest tertile, both diets were similarly effective. MetaboHealth remained unchanged.

**Conclusions:**

Combining metabotype with MetaboHealth may enhance personalized dietary strategies for fat loss in insulin‐resistant adults.

## 1 Introduction

The rising prevalence of insulin resistance (IR) in aging populations poses a tremendous challenge to health care systems worldwide [1, 2]. With aging, excess adipose tissue mass tends to accumulate viscerally and ectopically, thereby contributing to an increased risk of developing IR‐related diseases—the leading causes of morbidity and mortality in middle‐aged and older adults [3, 4]. Weight management interventions have been demonstrated to be moderately successful in reducing IR [5]. However, there is a large heterogeneity of response to lifestyle modifications, where not all individuals benefit [6, 7]. For this reason, the development of more personalized dietary strategies is essential to improve body composition and mitigate IR‐related risks effectively [8]. The manifestation of IR is not uniform; it can present as predominant muscle insulin resistance (MIR) or predominant liver insulin resistance (LIR), underscoring the need for personalized treatment strategies tailored to individual metabotypes [9, 10].

The PERSonalized Glucose Optimization Through Nutritional Intervention (PERSON) study previously investigated health effects of dietary interventions designed for individuals with either the MIR or LIR metabotypes [11]. Participants were assigned to one of two isocaloric dietary regimens: a low‐fat, high‐protein, high‐fiber (LFHP) diet or a high‐monounsaturated fat (HMUFA) diet [12, 13, 14]. Participants in the MIR group showed improvements in cardiometabolic outcomes, including peripheral insulin sensitivity, glucose homeostasis, serum triacylglycerol, and C‐reactive protein (CRP), when following the LFHP diet. Conversely, the LIR group benefited more from the HMUFA diet for these outcomes [15]. However, both diets had similar effects on body weight and body composition, irrespective of IR metabotype. This lack of differential effects suggests a potential for further refinement of the initial IR metabotype classifications by introducing additional grouping criteria.

The recently developed blood‐based ^1^H‐NMR metabolomics‐based MetaboHealth score (MH) provides a novel biomarker to assess individual immune‐metabolic health comprehensively [16, 17, 18]. The MetaboHealth score is derived from a study to predict all‐cause mortality in 44,168 individuals from 12 cohorts and is composed of 14 circulating metabolomic measures, including lipoprotein particle sizes, polyunsaturated fatty acid (PUFA) ratio, and concentrations of histidine, leucine, valine, albumin, glucose, lactate, isoleucine, phenylalanine, acetoacetate, and glycoprotein acetyls (GlycA)—a marker of inflammation [19]. Higher MetaboHealth scores indicate poorer immune‐metabolic health and an increased risk for mortality, frailty, and cognitive decline [16, 18, 20].

This study aimed to determine whether the MetaboHealth score, as a global indicator of immune‐metabolic health, can enhance the analysis of dietary intervention efficacy beyond the grouping of participants by tissue‐specific IR metabotype. Specifically, we explored how baseline MetaboHealth score tertiles were associated with dietary intervention‐induced changes in (i) cardiometabolic outcomes and (ii) body composition, including metrics of fat and lean mass [15]. By integrating this additional grouping, we seek to refine precision nutrition strategies.

## 2 Methods

### 2.1 Study Design

**FIGURE 1:**
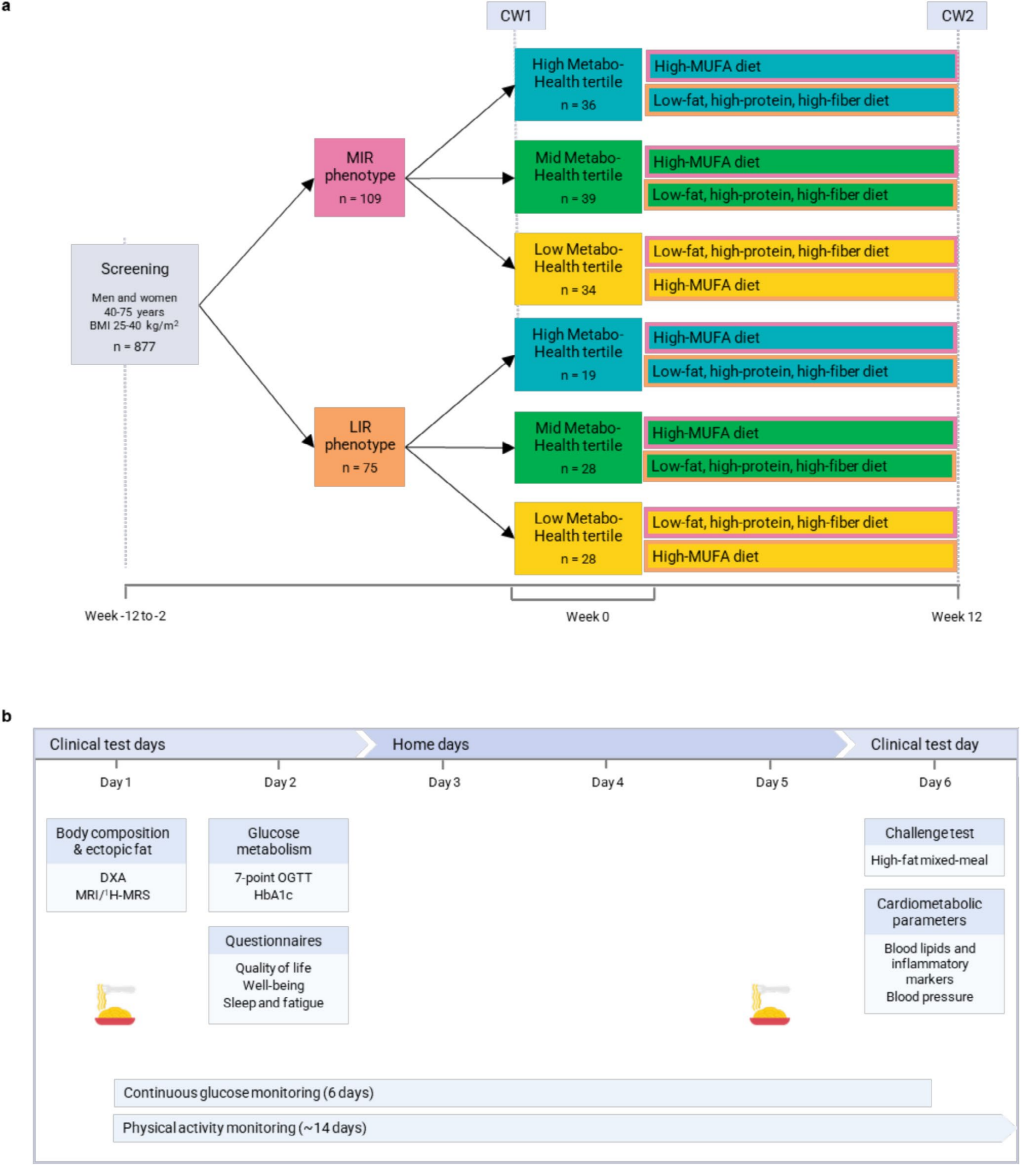
PERSON study design. (A) Participants were initially classified into two groups based on the predominant type of insulin resistance—muscle insulin resistance (MIR) or liver insulin resistance (LIR)—using a 7‐point oral glucose tolerance test (OGTT) at screening. After classification participants were further categorized into MetaboHealth tertiles—Low, Medium, or High—based on baseline ^1^H‐NMR metabolomics measurements. All participants shown in the subgroups represent the final analytical sample included in the study. Participants in all groups followed one of two dietary interventions during the study period: a high‐monounsaturated fatty acid (HMUFA) diet and a low‐fat, high‐protein, high‐fiber (LFHP) diet. The order in which participants followed each diet was randomized. allowing each phenotype to experience both diets during the intervention. (B) Clinical assessments and at‐home measurements were conducted at two time points: Week 0 (the start of the study) and Week 12 (the end of the intervention). These assessments included comprehensive evaluations of metabolic health, physical activity, and dietary adherence. Adapted from Trouwborst et al. [15]. [Color figure can be viewed at wileyonlinelibrary.com]

This study was part of the PERSON study, a two‐center, randomized, double‐blind, controlled trial designed to assess the effects of metabotype‐specific dietary interventions on cardiometabolic health in a middle‐aged and older population (Figure 1). A total of 242 men and women with overweight or obesity aged 40–75 years, with BMI of 25–40 kg/m^2^, participated in this study. Participants were screened using a 7‐point oral glucose tolerance test (OGTT) to determine the hepatic insulin resistance index (HIRI) and muscle insulin sensitivity index (MISI). These indices were used to identify participants with predominant MIR or LIR. Eligible participants were then randomly assigned to follow either an HMUFA or a LFHP diet for 12 weeks. Participants underwent extensive characterization at baseline and the end of the 12‐week intervention. The trial took place between May 2018 and November 2021 at Maastricht University Medical Center^+^ (MUMC^+^) and Wageningen University and Research (WUR) in the Netherlands, adhering to the principles of the Declaration of Helsinki. The study protocol received approval from the Medical Ethical Committee of MUMC^+^ (NL63768.068.17) and was registered at ClinicalTrials.gov (NCT03708419). Written informed consent was obtained from all participants.

### 2.2 Assessment of Tissue‐Specific Insulin Resistance (IR)

Tissue‐specific IR was assessed using a 7‐point OGTT, where participants consumed 75 g of glucose (200 mL solution, Novolab) within 5 min. Blood was sampled via an intravenous cannula at fasting (*t* = 0) and at 15–120 min post ingestion to measure plasma glucose and insulin. HIRI and MISI were calculated, with MISI optimized using a cubic spline method [11]. HIRI was derived from the 0–30 min glucose and insulin AUCs, while MISI was defined as the glucose decay rate (from peak to nadir) divided by mean insulin during the OGTT.

Glucose curves unsuitable for MISI calculation (e.g., peak at 120 min, flat curves, rebound) were visually inspected for classification. Both indices were validated against the hyperinsulinemic‐euglycemic clamp [10, 21]. Participants were categorized as “No MIR/LIR,” “MIR,” “LIR,” or “combined MIR/LIR” using tertile cutoffs from The Maastricht Study (DMS). The lowest MISI tertile defined MIR and the highest HIRI tertile defined LIR. Since LIR prevalence was lower in the PERSON study compared to DMS, the PERSON study median HIRI was used for subsequent classifications [11].

### 2.3 Dietary Intervention

The HMUFA diet provided 38% of energy from fat (20% MUFA, 8% PUFA, and 8% SFA), 42% from carbohydrates (30% polysaccharides; 3 g/MJ fiber), and 14% from protein. The LFHP diet consisted of 28% fat (10% MUFA, 8% PUFA, and 8% SFA), 42% carbohydrates (30% polysaccharides; > 4 g/MJ fiber), and 24% protein. Key products that participants were provided with for the HMUFA diet included olive oil, olives, and low‐fat margarine, while for the LFHP diet, these included low‐fat yogurt, reduced‐fat cheese, and a fiber supplement (2 g β‐glucan per 6 g). Participants were instructed to consume prescribed portions daily, with alcohol limited to one glass per day. Both diets were in line with the Dutch dietary guidelines [22].

Energy intake was personalized, ranging from 6 to 13 MJ/day, based on each participant's estimated needs, calculated from self‐reported dietary intake and physical activity. Weekly counseling sessions were provided to ensure diet adherence, monitor weight stability, and address any concerns. Participants received specific food products tailored to their assigned diet (HMUFA or LFHP), and adherence was monitored through detailed food logs, regular counseling sessions, and compliance checks throughout the 12‐week intervention. Dietary compliance was further assessed using three unannounced 24‐h recalls on two nonconsecutive weekdays and one weekend day via the mobile app *Traqq* [23]. In addition, plasma fatty acid profiles were analyzed by nuclear magnetic resonance metabolomics as objective biomarkers of SFA, MUFA, and PUFA consumption [24].

### 2.4 Measurements

Participants were extensively phenotyped at baseline and Week 12, during a characterization week that included clinical test days and at‐home data collection. Participants were instructed to refrain from alcohol and vigorous physical activity the day before and during the characterization weeks.

### 2.5 7‐Point Oral Glucose Tolerance Test (OGTT)

The 7‐point OGTT was conducted following the same procedures as the screening visit. Participants consumed a standardized low‐fat meal the evening before the test and remained fasted until the OGTT. The outcome, the disposition index, was calculated as: [Matsuda index * (AUC30 min insulin/AUC30 min glucose)], where AUC30 min is the area under the curve for insulin and glucose from baseline to 30 min. The Matsuda index was calculated with the following formula: [10,000 ÷ square root of [fasting plasma glucose (mg/dL) × fasting insulin (mU/L)] × [mean glucose (mg/dL) × mean insulin (mU/L)]], using glucose and insulin values of time points 0, 30, 60, 90, and 120 min. Additional indices including HOMA‐IR and HOMA‐β were calculated. WHO criteria were used to define glucose status, including normal glucose tolerance (NGT), impaired fasting glucose (IFG), impaired glucose tolerance (IGT), and type 2 diabetes (T2DM).

### 2.6 High‐Fat Mixed‐Meal Challenge Test and Biochemical Analysis

At least 4 days after the OGTT, participants underwent a high‐fat mixed‐meal challenge (HFMM) to assess postprandial glucose and lipid metabolism. Participants consumed the same low‐fat macaroni meal as the OGTT on the night before the visit. After an overnight fast, participants consumed a liquid HFMM (350 g, 2.8 MJ, and 64% fat), and blood samples were collected at fasting (*t* = 0) and at 30, 60, 90, 120, 180, and 240 min post consumption to measure triacylglycerol. CRP was measured in fasting plasma using a Luminex immunoassay performed by DSM Nutritional Products (Kaiseraugst, Switzerland). In addition, metabolomics profiles of fasted plasma samples were determined using the ^1^H‐NMR metabolomics‐based Nightingale Health platform [25, 26, 27].

### 2.7 Body Composition Analysis

Body composition was assessed by dual‐energy X‐ray absorptiometry (DXA) (MUMC+, Discovery A, Hologic; WUR, Lunar Prodigy, GE Healthcare). This included measures such as android fat percentage of total android mass, gynoid fat percentage of total gynoid mass, total fat percentage, total lean mass percentage, fat mass index (kg/m^2^), lean mass index (kg/m^2^), and appendicular lean mass index (kg/m^2^) [25, 26, 27]. More details on the study variables are available elsewhere [11].

### 2.8 MetaboHealth Score

The MetaboHealth score is a normalized composite of 14 biomarkers—total lipids in chylomicrons and extremely large VLDL (XXL‐VLDL‐L), total lipids in small HDL (S‐HDL‐L), mean diameter for VLDL particles (VLDL‐D), ratio of PUFA to total fatty acids (PUFA %), glucose, lactate, histidine, isoleucine, leucine, valine, phenylalanine, acetoacetate, albumin, and GlycA—derived from its association with all‐cause mortality in 44,168 individuals across 12 European cohorts [16]. It assesses cardiovascular, metabolic, and muscle health, with higher scores indicating a higher mortality risk. The MetaboHealth score was calculated as a weighted sum of 14 biomarker values [16], which were first natural log‐transformed and z‐standardized to reduce skewness using standard base function in R (version 4.2.3).

Primary analyses focused on participants in the lowest and highest baseline MetaboHealth tertiles, as these groups exhibit the most pronounced differences in cardiometabolic and body composition outcomes. The middle tertile was excluded to maximize contrast and improve interpretability of the results.

### 2.9 Statistical Analysis

**FIGURE 2:**
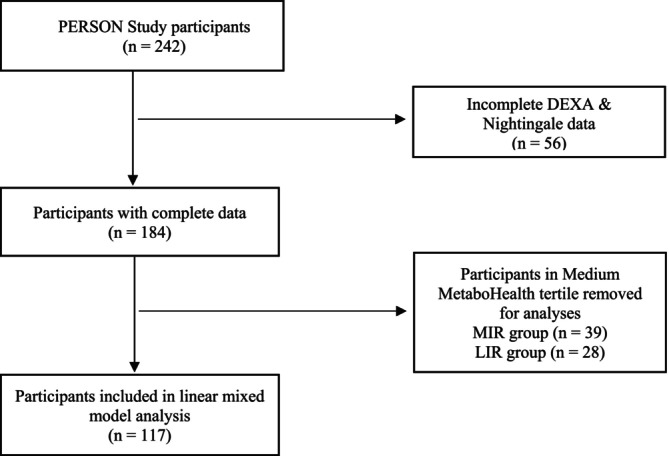
Flow diagram of the final included participants from the original PERSON study cohort.

We analyzed the outcomes of the intervention by contrasting the highest and lowest baseline MetaboHealth tertiles. Before analysis, participants with incomplete outcome data were excluded. The aim of our current post hoc analysis was to see whether further refinement of MIR and LIR with MetaboHealth would add to the additional health benefit. Analyses were therefore conducted using a complete‐case approach, thus including a sample size of 117 participants (excluding the medium tertiles from both metabotypes) with a complete dataset (Figure 2) [20]. The primary outcomes analyzed included the MetaboHealth score, MISI, and HIRI. The secondary outcomes included the cardiometabolic health outcomes HOMA‐IR, HOMA‐B, Matsuda index, disposition index, CRP, and triacylglycerides and DXA‐derived body composition metrics, such as android fat percentage, gynoid fat percentage, total fat percentage, fat mass index, lean mass index, and appendicular lean mass index.

For the mixed‐effects model analysis, traditional covariates included age, sex, and BMI. Explanatory variables of interest included metabotype (MIR or LIR), diet (HMUFA or LFHP), and the MetaboHealth score tertiles defined at baseline (low versus high). The coding is detailed in Table S1. The model included main effects for age, sex, BMI (only for models predicting the MetaboHealth score), time, diet, metabotype, and baseline MetaboHealth tertile. Interactions included combinations of time, diet, metabotype, and MetaboHealth tertile, with a random intercept for participant ID to account for repeated measures. The model is as follows:

$$\begin{array}{c} \text{Outcome}=\beta^{0}+\beta^{1}\cdot \mathrm{Age}+\beta^{2}\cdot \mathrm{Sex}+\beta^{3*}\cdot \mathrm{BMI}\;\;\\ \;+\beta^{4}\cdot \text{Time}+\beta^{5}\cdot \text{Diet}+\beta^{6}\cdot \text{Metabotype}\\ \;+\beta^{7}\cdot \text{MetaboHealth Tertile}+\beta^{8}\cdot \left(\text{Time}\cdot \text{Diet}\right)\\ \;+\beta^{9}\cdot \left(\text{Time}\cdot \text{Metabotype}\right)+\beta^{10}\cdot \left(\text{Time}\cdot \text{MetaboHealth Tertile}\right)\\ \;+\beta^{11}\cdot \left(\text{Diet}\cdot \text{Metabotype}\right)\;+\beta ₁₂\cdot \left(\text{Diet}\cdot \text{MetaboHealth Tertile}\right)\\ \;+\beta ₁₃\cdot \left(\text{Metabotype}\cdot \text{MetaboHealth Tertile}\right)\\ \;+\beta ₁₄\cdot \left(\text{Time}\cdot \text{Diet}\cdot \text{Metabotype}\right)\\ \;+\beta ₁₅\cdot \left(\text{Time}\cdot \text{Diet}\cdot \text{MetaboHealth Tertile}\right)\\ \;+\beta ₁₆\cdot \left(\text{Time}\cdot \text{Metabotype}\cdot \text{MetaboHealth Tertile}\right)\\ \;+\beta ₁₇\cdot \left(\text{Diet}\cdot \text{Metabotype}\cdot \text{MetaboHealth Tertile}\right)\\ \;+\;\beta ₁₈\cdot \left(\text{Time}\cdot \text{Diet}\cdot \text{Metabotype}\cdot \text{MetaboHealth Tertile}\right)\\ \;+\left(1\;|\;\mathrm{ID}\right). \end{array}$$

The inclusion of BMI as a covariate was restricted to models where the MetaboHealth score was the outcome, as the DXA‐derived body composition measures inherently adjusted for variations in body composition. The mixed‐effects models were built using the lme4 package followed by the estimation of marginal means with the emmeans package [28, 29]. EMMs were derived from the fitted models to help interpretation and visualization of significant higher‐order interactions, particularly the four‐way interaction between baseline Metabotype, baseline MetaboHealth tertile (low vs high), Diet, and Time. Additional packages included lme4, lmerTest, ggplot2, dyplr, and tidyr in R version 4.2.3 to visualize predictive models for the four‐way interactions [30, 31, 32, 33]. To account for multiple comparisons, *p* values were adjusted using the false discovery rate (FDR) Benjamini–Hochberg method, and results with an adjusted *p* < 0.05 were considered statistically significant.

## 3 Results

### 3.1 Stratifying Baseline Metabotypes by MetaboHealth Tertiles Refines Differences in Health Parameters Between the Groups

We investigated the baseline characteristics of the groups combining metabotype and MetaboHealth tertiles. This resulted in six groups—LIR.LowMetaboHealth (*n* = 28), MIR.LowMetaboHealth (*n* = 34), LIR.MediumMetaboHealth (*n* = 28), MIR.MediumMetaboHealth (*n* = 39), LIR.HighMetaboHealth (*n* = 19), and MIR.HighMetaboHealth (*n* = 36). We investigated the differences between these groups with cardiometabolic health and body composition outcomes. We observed differences in 13 out of 15 baseline characteristics between the groups indicating that adding the MetaboHealth to metabotype stratification further refines the group differences in key characteristics (Table 1).

**TABLE 1:** Baseline characteristics of the PERSON cohort participants selected in groups of metabotype and MetaboHealth combined.

	Metabotype:MetaboHealthtertile grouping						Significance	All (*N* = 184)
	MIR:MetaboHealth tertile			LIR:MetaboHealth tertile				
	Low (*N* = 34)	Medium (*N* = 39)	High (*N* = 36)	Low (*N* = 28)	Medium (*N* = 28)	High (*N* = 19)		
							*p*	
Cardiometabolic health variables								
MetaboHealth score; mean (SD)	−0.3 (0.1)^a^	0.0 (0.1)^b^	0.3 (0.2)^c^	−0.3 (0.1)^a^	0.0 (0.1)^b^	0.3 (0.1)^c^	**8.33 × 10** ^**−53**^	0.0 (0.3)
MISI (AU); mean (SD)	0.13 (0.08)^ab^	0.15 (0.08)^ab^	0.13 (0.09)^a^	0.18 (0.12)^ab^	0.12 (0.06)^ab^	0.20 (0.11)^b^	**0.018**	0.15 (0.09)
HIRI (AU); mean (SD)	383.9 (180.5)^ab^	337.8 (267.4)^a^	396.2 (204.2)^abc^	598.3 (426.7)^c^	583.4 (311.2)^bc^	560.0 (262.6)^abc^	**1.05 × 10** ^**−04**^	457.7 (296.6)
HOMA‐IR (AU); mean (SD)	1.64 (0.55)^a^	1.64 (0.81)^a^	2.17 (1.44)^ab^	1.77 (0.68)^a^	1.93 (0.92)^ab^	2.77 (2.34)^b^	**0.007**	1.92 (1.2)
HOMA‐β (AU); mean (SD)	81.7 (25.3)	77 (28.9)	87.1 (32.5)	78 (23.7)	86.6 (39.6)	84.6 (23.8)	0.629	82.2 (29.6)
Matsuda index (AU); mean (SD)	5.0 (1.8)^ab^	6.1 (3.2)^a^	4.7 (2)^ab^	5.2 (2.1)^ab^	4.6 (2.3)^ab^	4.0 (2.0)^ab^	**0.014**	5 (2.4)
Disposition index (AU); mean (SD)	160.4 (64)^ab^	155.1 (65.6)^ab^	148.1 (86.3)^a^	209.5 (112.4)^b^	182.9 (87.3)^ab^	145.0 (90.4)^ab^	**0.035**	166.2 (85.6)
CRP (mg/L); mean (SD)	1.22 (1.02)^ab^	1.52 (1.24)^abc^	2.39 (1.58)^c^	0.76 (0.18)^a^	1.74 (1.51)^abc^	2.07 (1.54)^bc^	**6.79 × 10** ^**−06**^	1.61 (1.43)
TAG (mg/L); mean (SD)	1.45 (0.58)	1.31 (0.36)	1.42 (0.67)	1.45 (0.62)	1.60 (0.73)	1.50 (0.54)	0.633	1.44 (0.59)
Body composition variables								
Android fat %; mean (SD)	44.1 (6.8)^ab^	45.8 (7.7)^ab^	47.5 (6.0)^a^	42 (6.4)^b^	44.7 (6.6)^ab^	45.6 (5.5)^ab^	**1.26 × 10** ^**−08**^	45.1 (6.8)
Gynoid fat %; mean (SD)	36.8 (8.9)^ab^	39.6 (11)^ab^	40.5 (8.8)^a^	33.9 (8.7)^b^	38.0 (6.9)^ab^	40.0 (7.8)^ab^	**1.23 × 10** ^**−09**^	38.2 (9.1)
Total fat %; mean (SD)	35.5 (6.5)^ab^	37.7 (8.9)^ab^	39.8 (7.2)^a^	33.6 (7.3)^b^	37.5 (6.4)^ab^	39.7 (6.7)^ab^	**8.21 × 10** ^**−11**^	37.2 (7.5)
Fat mass index (kg/m^2^); mean (SD)	10.4 (2.5)^a^	11.1 (3.3)^ab^	12.0 (3.3)^ab^	10.0 (2.8)^a^	11.5 (3.0)^ab^	13.1 (3.7)^b^	**3.34 × 10** ^**−06**^	166.9 (55.1)
Lean mass index (kg/m^2^); mean (SD)	19.1 (2.3)^ab^	16.9 (1.5)^c^	17.5 (2.2)^ac^	19.3 (2.2)^b^	18.9 (2.0)^ab^	18.6 (2.2)^abc^	**9.09 × 10** ^**−11**^	11.2 (3.2)
Appendicular mass index (kg/m^2^); mean (SD)	8.4 (1.1)^ab^	7.2 (0.8)^a^	7.3 (1)^ab^	8.5 (1.4)^b^	8.2 (0.9)^ab^	7.9 (1.2)^ab^	**6.79 × 10** ^**−14**^	18.4 (2.2)
VAT area (cm^2^)	165.8 (58.4)	153.3 (54.9)	177 (59.4)	168.4 (45)	168.6 (54.2)	173.4 (57.2)	0.098	
Traditional covariates								
Age (years); mean (SD)	61.4 (7.5)	59.8 (8.4)	60.3 (8.1)	60.8 (6.7)	58 (6.9)	59.6 (8.8)	0.774	60.1 (7.91)
Sex (M); count (% total)	16 (44.1%)	13 (35.9%)	13 (36.1%)	15 (60.7%)	9 (39.3%)	11 (42.1%)	—	78 (42.4%)
BMI; mean (SD)	29.3 (2.8)	29 (3)	29.7 (4)	29.4 (2.9)	30.5 (4)	32.6 (4.5)	0.132	25.9 (4.0)

*Note*: One‐way ANOVA and Tukey HSD tests were carried out to determine mean differences across six levels of metabotype and MetaboHealth tertiles (Low‐Medium‐High). The letters (a, b, c, etc.) indicate Tukey HSD groupings, where shared letters mean no significant difference, and different letters indicate significant differences (*p* < 0.05). Adjusted *p* < 0.05 were considered statistically significant (in bold).

Abbreviations: CRP, C‐reactive protein; HIRI, hepatic insulin resistance index; MISI, muscle insulin sensitivity index; *n* represents the number of participants with a complete dataset at both baseline and endline; TAG, triacylglycerides; VAT, visceral adipose tissue.

Despite similar ages and BMI levels across groups (*p* = 0.774 and *p* = 0.132, respectively), sex distribution varied, with the highest percentage of males in LIR.LowMetaboHealth (60.7%) and the lowest in MIR.MediumMetaboHealth (35.9%) groups. Concerning body composition measures, individuals with predominant MIR consistently had higher android, gynoid, and total fat percentages compared to those with predominant LIR (*p* < 0.001 for all). The fat mass index was significantly higher in both MIR.HighMetaboHealth and LIR.HighMetaboHealth groups compared to the other groups (*p* = 3.34 × 10^−06^), while lean mass indices were lower in the MIR.HighMetaboHealth tertiles (*p* = 9.09 × 10^−11^). Moreover, individuals in the LIR.HighMetaboHealth and MIR.HighMetaboHealth tertiles had higher CRP levels compared to those in the lower tertiles (*p* = 6.79 × 10^−06^), demonstrating that the MetaboHealth score reflects overall immune‐metabolic health.

### 3.2 In Response to the Intervention, the Refined IR Metabotype‐MetaboHealth Combined Phenotypes Reveal Differences in Fat Loss, but Not Cardiometabolic Health Outcomes

For the outcomes, we did not find any significant four‐way interactions for MetaboHealth score, disposition index, MISI, HIRI, HOMA‐IR, HOMA‐β, Matsuda index, and CRP, which were not significant (Table S3).

Next, we analyzed whether grouping MetaboHealth tertiles refined response differences between the two diets for MIR and LIR metabotypes concerning body composition changes as outcomes. A four‐way interaction model was used to investigate differences in response based on the six groups. Significant four‐way interactions between baseline metabotype and MetaboHealth tertile, diet, and time emerged, particularly in fat percentage outcomes. Table 2 presents regression coefficients for all terms, with particular focus on the three‐ and four‐way interaction of MIR and LIR metabotypes with the highest and lowest MetaboHealth tertiles across both HMUFA and LFHP diets. Individuals with the MIR metabotype following the LFHP diet had significant reductions in android fat (*β* = −0.71, 95% CI [−1.33, −0.38], *q* = 4.66 × 10^−14^), gynoid fat (*β* = −0.68, 95% CI [−0.98, −0.25], *q* = 0.002), and total fat percentages (*β* = −0.46, 95% CI [−0.80, −0.12], *q* = 0.001) and fat mass index (*β* = −0.19, 95% CI [−0.29, −0.09], *q* = 0.002), with narrow CIs confirming the robustness of these effects. Similarly, MIR individuals in the high MetaboHealth tertile at baseline following the LFHP diet experienced a slight but significant reduction in total fat percentage (*β* = −0.11, 95% CI [−0.27, −0.05], *q* = 0.048). These MIR high MetaboHealth participants showed a similar significant decrease in android fat percentage across the intervention regardless of diet (*β* = −0.16, 95% CI [−0.34, −0.06], *q* = 0.019). The significant four‐way interaction between time, diet, metabotype, and MetaboHealth tertile revealed differential intervention effects on android, gynoid, and total fat percentages and fat mass index (*β* = 0.28, 95% CI [0.13, 0.54], *q* = 0.003; *β* = 0.28, 95% CI [0.07, 0.39], *q* = 0.008; *β* = 0.17, 95% CI [0.02, 0.31], *q* = 0.013; and *β* = 0.07, 95% CI [0.02, 0.11], *q* = 0.008, respectively), with CIs consistently excluding zero, supporting significant effects. Changes in lean mass index, appendicular lean mass index, and VAT area following the intervention were not significantly different between groups (Table S2).

**TABLE 2:** Effect sizes showing the association between the baseline combined metabotype‐MetaboHealth tertile groups and body composition changes in response to the intervention.

Model variable	Android fat %		Gynoid fat %		Total fat %		Fat mass index (kg/m^2^)	
	Estimate	Adjusted *p*	Estimate	Adjusted *p*	Estimate	Adjusted *p*	Estimate	Adjusted *p*
(Intercept)	47.05	**4.66 × 10** ^**−14**^	43.89	**9.11 × 10** ^**−15**^	40.27	**5.09 × 10** ^**−16**^	13.48	**1.24 × 10** ^**−07**^
Age	−0.09	0.518	−0.07	0.580	−0.06	0.580	−0.05	0.271
Sex (male)	−4.21	**0.006**	−13.78	**1.33 × 10** ^**−20**^	−10.78	**3.28 × 10** ^**−18**^	−3.35	**6.49 × 10** ^**−08**^
Time	−0.27	**0.021**	−0.22	0.098	−0.21	**0.008**	−0.09	**0.013**
Diet (LFHP)	2.45	0.731	0.87	0.885	2.50	0.612	1.07	0.722
Metabotype (MIR)	0.85	0.885	1.19	0.841	2.46	0.610	1.67	0.530
MetaboHealthTertile.High	1.88	0.314	1.58	0.371	2.62	**0.035**	1.57	**0.019**
Time:dietLFHP	0.19	0.287	0.38	**0.023**	0.26	**0.019**	0.10	**0.037**
Time:MetabotypeMIR	0.43	**0.003**	0.37	**0.021**	0.25	0.018	0.09	**0.040**
dietLFHP:MetabotypeMIR	1.38	0.884	0.31	0.971	−3.99	0.549	−2.30	0.535
Time:MHTertileHigh	0.05	0.510	0.03	0.669	0.03	0.518	0.01	0.702
dietLFHP:MHTertileHigh	−0.99	0.758	0.62	0.840	−1.26	0.610	−0.80	0.549
MetabotypeMIR:MHTertileHigh	0.39	0.885	−0.06	0.976	−1.19	0.580	−1.22	0.204
Time:dietLFHP:MetabotypeMIR	−0.71	**3.41 × 10** ^**−04**^	−0.68	**0.002**	−0.46	**0.001**	−0.19	**0.002**
Time:dietLFHP:MHTertileHigh	−0.12	0.170	−0.16	0.058	−0.11	**0.048**	−0.04	0.098
Time:MetabotypeMIR:MHTertileHigh	−0.16	**0.019**	−0.12	0.106	−0.08	0.098	−0.03	0.204
dietLFHP:MetabotypeMIR:MHTertileHigh	−0.69	0.879	−1.18	0.750	1.81	0.564	1.31	0.400
Time:dietLFHP:MetabotypeMIR:MHTertileHigh	0.28	**0.003**	0.28	**0.008**	0.17	**0.013**	0.07	**0.018**

*Note*: Regression coefficients of mixed linear models exploring the four‐way interaction of MIR and LIR metabotypes with highest and lowest MetaboHealth (MH) tertiles and two diets across the intervention. FDR‐adjusted *p*‐values are presented. Adjusted *p* < 0.05 were considered statistically significant (in bold).

### 3.3 Visualization of the Four‐Way Interaction Effects on Metabotypes and Diet Reveals Further Differences in Android and Gynoid Fat Percentage Outcomes Across Low and High MetaboHealth Tertiles

The estimated marginal means were used to visualize the significant four‐way interaction between baseline metabotype and MetaboHealth tertile (contrasting low versus high), applied at baseline, diet, and time concerning decreases in android, gynoid, and total fat percentages and fat mass index across the intervention (Figures 3, 4, 5, 6). Additional graphs for other body composition and metabolic outcomes that did not demonstrate significant four‐way interactions are presented in Figures S1–S12, with all EMM values detailed in Tables S4 and S5.

**FIGURE 3:**
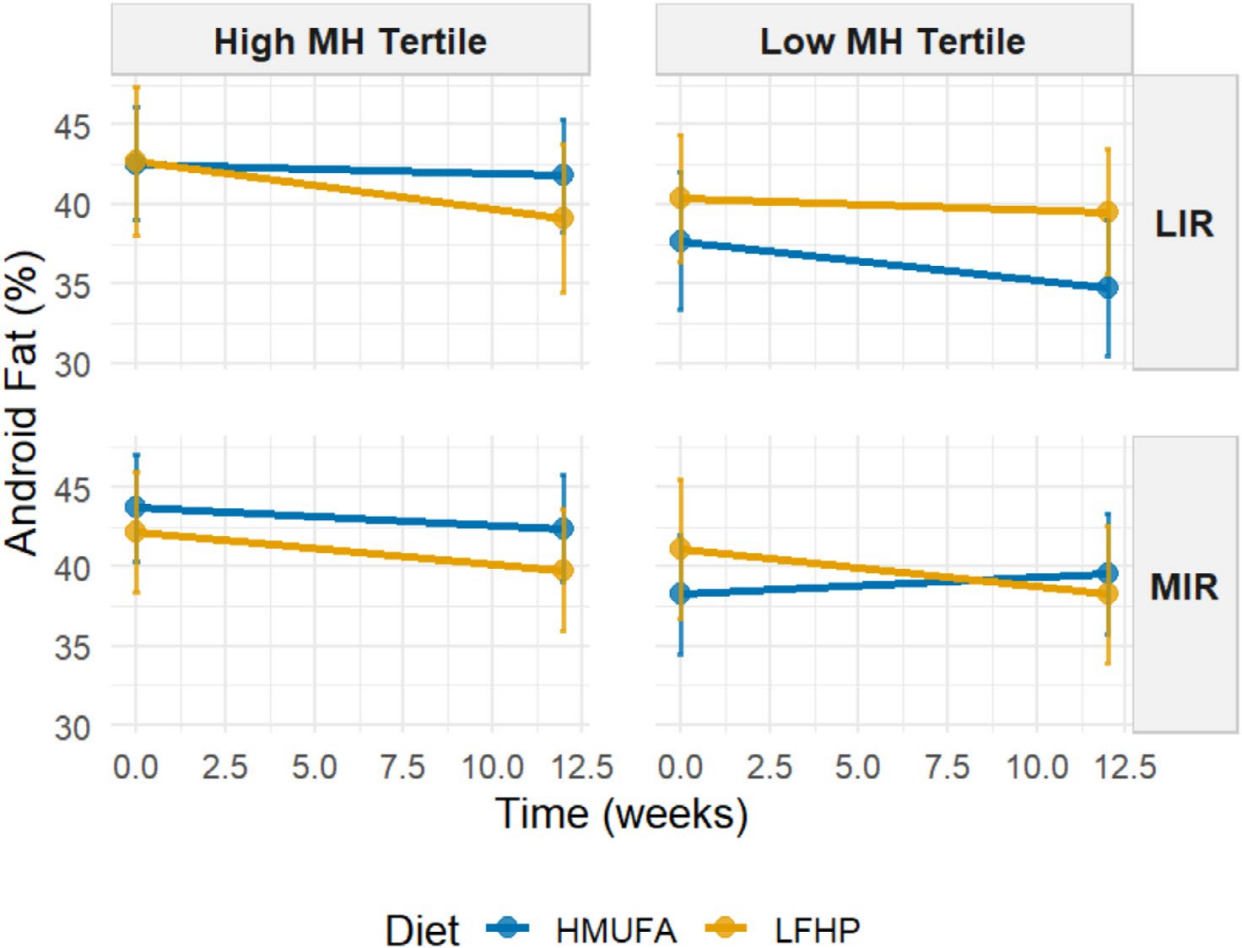
Estimated marginal means for android fat percentage by IR metabotype and MH tertiles across diets. A 2 × 2 matrix shows estimated total fat percentage grouped by IR metabotype (MIR vs. LIR) and MH tertiles (high vs. low). The low‐fat, high‐protein, high‐fiber (LFHP) diet is in yellow and the high‐monounsaturated fat (HMUFA) diet in blue. The range at each point represents the upper and lower CIs. LIR.Low (*n* = 28). LIR.High (*n* = 19). MIR.Low (*n* = 34). MIR.High (*n* = 36). [Color figure can be viewed at wileyonlinelibrary.com]

**FIGURE 4:**
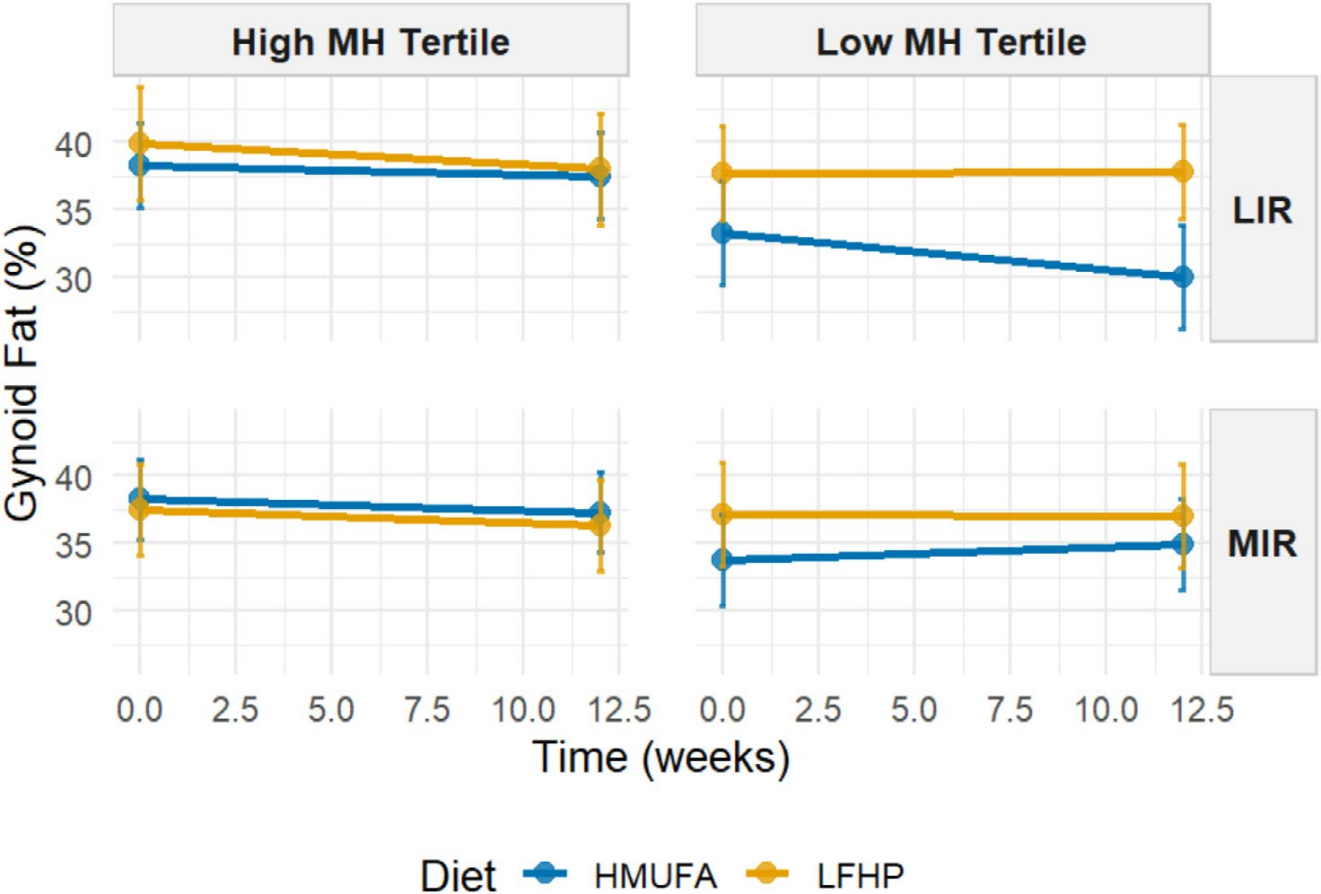
Estimated marginal means for gynoid fat percentage by IR metabotype and MH tertiles across diets. A 2 × 2 matrix shows estimated total fat percentage grouped by IR metabotype (MIR vs. LIR) and MH tertiles (high vs. low). The low‐fat, high‐protein, high‐fiber (LFHP) diet is in yellow and the high‐monounsaturated fat (HMUFA) diet in blue. The range at each point represents the upper and lower CIs. LIR.Low (*n* = 28). LIR.High (*n* = 19). MIR.Low (*n* = 34). MIR.High (*n* = 36). [Color figure can be viewed at wileyonlinelibrary.com]

**FIGURE 5:**
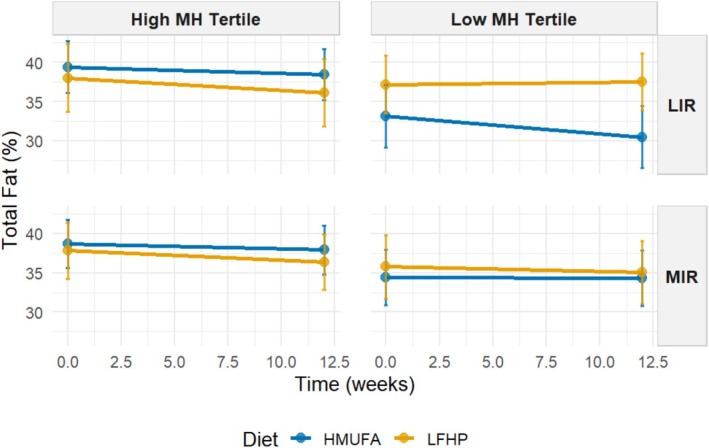
Estimated marginal means for total fat percentage by IR metabotype and MH tertiles across diets. A 2 × 2 matrix shows estimated total fat percentage grouped by IR metabotype (MIR vs. LIR) and MH tertiles (high vs. low). The low‐fat, high‐protein, high‐fiber (LFHP) diet is in yellow and the high‐monounsaturated fat (HMUFA) diet in blue. LIR.Low (*n* = 28). LIR.High (*n* = 19). MIR.Low (*n* = 34). MIR.High (*n* = 36). [Color figure can be viewed at wileyonlinelibrary.com]

**FIGURE 6:**
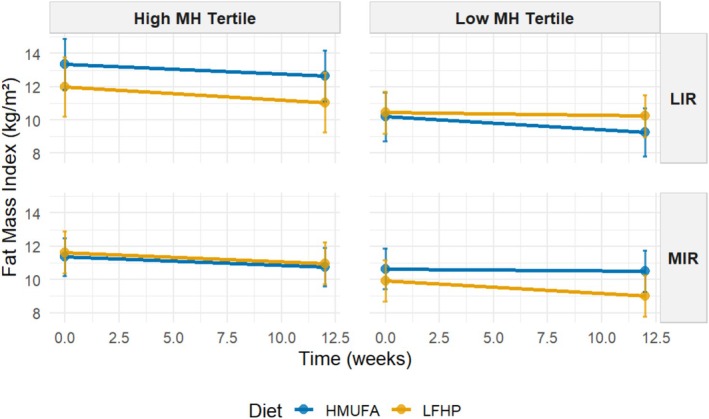
Estimated marginal means for fat mass index by IR metabotype and MH tertiles across diets. A 2 × 2 matrix shows estimated total fat percentage grouped by IR metabotype (MIR vs. LIR) and MH tertiles (high vs. low). The low‐fat, high‐protein, high‐fiber (LFHP) diet is in yellow and the high‐monounsaturated fat (HMUFA) diet in blue. LIR.Low (*n* = 28). LIR.High (*n* = 19). MIR.Low (*n* = 34). MIR.High (*n* = 36). [Color figure can be viewed at wileyonlinelibrary.com]

The LIR.LowMetaboHealth group experienced significant reductions in android fat on the HMUFA diet, while the LFHP diet showed no change. The LIR.HighMetaboHealth group showed reductions with both diets. In contrast, MIR.LowMetaboHealth participants had an increase in android fat on HMUFA but a moderate fat loss on LFHP, while participants in MIR.HighMetaboHealth benefited from reductions across both diets. For gynoid fat, LIR.LowMetaboHealth participants decreased on HMUFA but increased on LFHP, whereas the LIR.HighMetaboHealth group lost gynoid fat modestly with both diets. MIR.LowMetaboHealth participants maintained stable levels on LFHP but experienced an increase in HMUFA, and MIR.HighMetaboHealth participants saw slight losses regardless of diet. In terms of total fat percentage, LIR.LowMetaboHealth participants showed decreases on HMUFA and a slight reduction on LFHP, with the LIR.HighMetaboHealth group also decreasing on LFHP. MIR.LowMetaboHealth participants had minor changes on both diets, while MIR.HighMetaboHealth participants demonstrated reductions from both dietary approaches. Finally, regarding fat mass index, LIR.LowMetaboHealth participants benefited from HMUFA and the LIR.HighMetaboHealth group showed improvements from both diets. The MIR.LowMetaboHealth group found LFHP slightly more effective, while MIR.HighMetaboHealth participants experienced similar enhancements from both dietary interventions.

In summary, the results show that individuals in high MetaboHealth tertiles (those with poorer immune‐metabolic health status) experienced reductions in android, gynoid, and total fat percentage and fat mass index regardless of metabotype and diet. At low MetaboHealth tertiles (those with relatively better immune‐metabolic health status), the HMUFA diet largely benefitted individuals with the LIR metabotype, while the LFHP diet reduced android and gynoid fat in the MIR group.

## 4 Discussion

This study aimed to understand how personalized dietary interventions grouped by metabotype, specifically MIR and LIR, and MetaboHealth tertiles affect cardiometabolic health outcomes and body composition in a subset of 184 middle‐aged and older adults with complete data who participated in the PERSON study [15]. Stratifying for MetaboHealth did not refine the cardiometabolic outcomes of the study. Our findings did show, however, that individuals classified in the higher MetaboHealth score tertile (reflecting poorer immune‐metabolic health) demonstrate fat loss across both dietary regimens, while individuals in the lower MetaboHealth tertiles (reflecting better immune‐metabolic health) exhibit improvement in android–upper‐body and abdominal fat—and gynoid–lower‐body fat—percentages when on the LFHP diet and expressing the MIR phenotype or when on the HMUFA diet and expressing the LIR phenotype. This study emphasizes that precision nutrition strategies can be refined by synergistic indicators of metabolic health status in managing body weight control effectively.

To the best of our knowledge, this is the first study to explore the interaction between IR metabotypes and MetaboHealth tertiles to understand how middle‐aged and older adults with tissue‐specific IR respond to a personalized dietary intervention. Our findings indicate that the MetaboHealth score is an indicator of metabolic health synergistic to IR metabotypes [26, 34]. While the classification of tissue‐specific IR currently requires a 7‐point OGTT and is therefore mainly applied in research settings, it provides physiologically meaningful and validated indices of tissue‐specific IR and may form the basis for simplified diagnostic tools in broader clinical applications in the future. The phenotypes of MIR and LIR, defined by a single OGTT, are characterized by a robust and reproducible metabolome, lipidome, abdominal adipose tissue transcriptome, and systemic inflammatory profiles, representing distinct etiologies toward cardiometabolic disease. Dietary macronutrient modulation tailored to these phenotypes has been shown to considerably enhance cardiometabolic health [11]. Importantly, these phenotypes are already present in the overweight, normal glucose tolerant state—where 76% of individuals remain normoglycemic yet display early metabolic perturbations such as elevated waist circumference, cholesterol, and body fat percentage, even before the onset of impaired fasting glucose or impaired glucose tolerance [15]. Thus, this classification provides clinically meaningful phenotypes that may form the basis for precision nutrition strategies and simplified diagnostic tools in broader clinical application, showing that refining this classification with the MetaboHealth score may additionally favorably contribute to body composition profiles.

The PERSON study was initially set to improve cardiometabolic health parameters with insulin sensitivity as a primary outcome. The previous report as the current one showed that metabotypes indicated specific cardiometabolic benefits of the LFHP diet for the MIR group and the HMUFA diet for the LIR group, including measures of insulin sensitivity, triglycerides, and CRP [15], but no differential changes were observed in body composition. Apparently the additional baseline grouping by MetaboHealth score tertiles indicates changes in aspects of metabolic health other than cardiometabolic outcomes, such as body composition. Individuals in the highest MetaboHealth tertile experienced modest fat loss, irrespective of metabotype or diet intervention. This observation highlights how middle‐aged and older adults with IR, unhealthier cardiometabolic health profiles, and similar higher fat mass percentages at baseline, compared to those with healthier cardiometabolic health profiles, benefit from either of the two HMUFA or LFHP diets used in the PERSON study. In contrast, individuals with MIR in the lower MetaboHealth tertiles benefited significantly more from the LFHP diet, emphasizing the importance of global and tissue‐specific IR‐related metabolic health status of individuals at baseline [35, 36]. This refined, synergistic approach enhances our ability to predict which dietary approach—LFHP or HMUFA—will be more beneficial for middle‐aged and older adults to elicit modest fat mass loss over 3 months, rather than changing IR in this time frame. This also demonstrates that dietary interventions are not merely one‐size‐fits‐all but should be informed by a comprehensive understanding of an individual's cardiometabolic health profile [37].

The mechanisms underlying the observed responses to dietary interventions likely involve key interactions between dietary composition, insulin signaling pathways, and changes in body composition [35, 36, 37]. In individuals with LIR, the HMUFA diet, rich in monounsaturated fatty acids (MUFA), may provide specific benefits by improving hepatic lipid metabolism and reducing inflammation [38, 39]. Previous research suggests that MUFA may usher body fat loss specifically via the activation of AMP‐activated protein kinase signaling [40, 41]. Overall, MUFA enhance insulin sensitivity, optimize lipid profiles, and decrease the accumulation of harmful lipotoxic intermediates that can disrupt metabolic function [42, 43, 44]. Additionally, individuals with MIR following the LFHP diet may ensure higher protein intake, positively influencing insulin signaling and likely promoting the activation of pathways involved in glucose metabolism and muscle maintenance [45, 46]. This interplay may ensure effective nutrient utilization, facilitate energy balance, and promote fat oxidation while preventing excessive fat storage [47, 48]. By incorporating tailored dietary strategies, it may be possible to optimize health outcomes, possibly through these complex mechanistic pathways. To predict the response of individuals to dietary interventions requires more knowledge of metabolic status, overall health, age, sex, and related factors and includes complete analyses of body composition to understand the distribution of fat loss, and whether lean mass is preserved, across dietary interventions.

While our study demonstrated minor android, gynoid, total fat percentage, and fat mass indices loss with both dietary interventions, we did not observe improvements in the MetaboHealth score upon the intervention. It is important to note that the score was initially created as a risk indicator to predict long‐term (5–10 years) mortality and was found to indicate older individuals' global health status predicting decline of muscle, cognitive health, and frailty [18, 20]. This study shows that the MetaboHealth score as an outcome may not be particularly sensitive to short‐term dietary changes and accompanying metabolic health improvements in individuals with IR in this cohort. We, therefore, conclude that, although MetaboHealth effectively groups participants in tertiles at baseline, it may not adequately capture the nuances of cardiometabolic health changes in IR by short‐term dietary interventions. For the MetaboHealth score to change, major changes in its immune‐metabolic components would have to occur, which were not observed following the HMUFA or LFHP diets. Other short‐term interventions for individuals above 60 years of age, including ones in which diet and physical exercise are combined, are being explored in parallel, showing that especially interventions that change low‐grade inflammation appear to be recorded by the MetaboHealth score. Although it is not necessarily the most sensitive monitor for such effects, MetaboHealth does show improvement in global health [49, 50].

The strengths of this study include its robust design as part of a randomized controlled trial, the grouping of participants based on detailed metabolic profiles, and the use of well‐validated methods, such as DXA for body composition assessment and high‐throughput targeted metabolomic analysis. The grouping into MetaboHealth score tertiles allows for a more nuanced understanding of the impact of body composition on cardiometabolic health and its influence on dietary responses. However, there are limitations to consider. Firstly, subgroup sample sizes were imbalanced. The analyzed subgroups represent participants in the lowest and highest MetaboHealth tertiles combined with their respective metabotype (MIR or LIR). Consequently, some subgroups were relatively small (e.g., LIR.High *n* = 19 versus MIR. High *n* = 36), which reduces statistical power, particularly for detecting higher‐order interactions. Although the total sample size was 117 participants after excluding the medium tertiles, stratification limited the ability to draw definitive conclusions from three‐ or four‐way mixed model interactions. Replication in larger and more diverse cohorts will be necessary to confirm and extend these findings. Secondly, we used a complete‐case approach, as missing data precluded an intention‐to‐treat analysis, which may limit generalizability and introduce bias. Thirdly, the relatively short duration of the dietary interventions may not capture long‐term metabolic adaptations. Future studies should investigate the sustainability of these dietary approaches over extended periods. Additionally, the reliance on specific dietary regimens may not encompass the broader spectrum of dietary patterns beneficial for diverse populations, limiting the generalizability of our findings to other dietary patterns. Future research should explore the MetaboHealth score and other novel metabolomics or proteomics biomarkers or composite scores to monitor the response to lifestyle interventions.

In conclusion, our findings suggest that personalized dietary strategies for middle‐aged and older adults with IR can be more effective when considering both the specific metabolic phenotype and the broader MetaboHealth score defined in tertiles at baseline. This may indicate that adding global markers of immune‐metabolic health to disease‐specific ones when investigating responses of adults to interventions may provide outcome benefits. By integrating MetaboHealth score grouping into personalized dietary approaches, we offer valuable insights into optimizing body compositional health outcomes in insulin‐resistant individuals. Future interventions using a combination of personalized dietary regimens offer a comprehensive approach to managing IR and improving overall physical and metabolic health.

## Author Contributions

J.M.M.: conceptualized, analyzed, drafted, and finalized the manuscript. F.A.B., A.U., G.B.H., M.B., L.A., E.E.B., P.E.S.: helped conceptualize and revise the manuscript. A.G., G.H.G., and J.D.: revised and supported the manuscript.

## Funding

This work is funded by the VOILA Consortium (ZonMw 457001001), which had no role in the design and conduct of the study; collection, management, analysis, and interpretation of the data; and preparation, review, or approval of the manuscript.

## Conflicts of Interest

The authors declare no conflicts of interest.

## Supporting information


**Figure S1:** Estimated marginal means for lean mass index by IR metabotype and MH tertiles across diets. A 2 × 2 matrix shows estimated total fat percentage grouped by IR metabotype (MIR vs. LIR) and MH tertiles (high vs. low). The low‐fat. high‐protein. high‐fiber (LFHP) diet is in yellow and the high‐monounsaturated fat (HMUFA) diet in blue. LIR.Low (*n* = 28). LIR.High (*n* = 19). MIR.Low (*n* = 34). MIR.High (*n* = 36).
**FIgure S2:** Estimated marginal means for appendicular lean mass index by IR metabotype and MH tertiles across diets. A 2 × 2 matrix shows estimated total fat percentage grouped by IR metabotype (MIR vs. LIR) and MH tertiles (high vs. low). The low‐fat. high‐protein. high‐fiber (LFHP) diet is in yellow and the high‐monounsaturated fat (HMUFA) diet in blue. LIR.Low (*n* = 28). LIR.High (*n* = 19). MIR.Low (*n* = 34). MIR.High (*n* = 36).
**FIgure S3:** Estimated marginal means for VAT by IR metabotype and MH tertiles across diets. A 2 × 2 matrix shows estimated total fat percentage grouped by IR metabotype (MIR vs. LIR) and MH tertiles (high vs. low). The low‐fat. high‐protein. high‐fiber (LFHP) diet is in yellow and the high‐monounsaturated fat (HMUFA) diet in blue. LIR.Low (*n* = 28). LIR.High (*n* = 19). MIR.Low (*n* = 34). MIR.High (*n* = 36).
**FIgure S4:** Estimated marginal means for MetaboHealth score by IR metabotype and MH tertiles across diets. A 2 × 2 matrix shows estimated total fat percentage grouped by IR metabotype (MIR vs. LIR) and MH tertiles (high vs. low). The low‐fat. high‐protein high‐fiber (LFHP) diet is in yellow and the high‐monounsaturated fat (HMUFA) diet in blue. LIR.Low (*n* = 28). LIR.High (*n* = 19). MIR.Low (*n* = 34). MIR.High (*n* = 36).
**FIgure S5:** Estimated marginal means for MISI by IR metabotype and MH tertiles across diets. A 2 × 2 matrix shows estimated total fat percentage grouped by IR metabotype (MIR vs. LIR) and MH tertiles (high vs. low). The low‐fat. high‐protein high‐fiber (LFHP) diet is in yellow and the high‐monounsaturated fat (HMUFA) diet in blue. LIR.Low (*n* = 28). LIR.High (*n* = 19). MIR.Low (*n* = 34). MIR.High (*n* = 36).
**FIgure S6:** Estimated marginal means for HIRI by IR metabotype and MH tertiles across diets. A 2 × 2 matrix shows estimated total fat percentage grouped by IR metabotype (MIR vs. LIR) and MH tertiles (high vs. low). The low‐fat. high‐protein high‐fiber (LFHP) diet is in yellow and the high‐monounsaturated fat (HMUFA) diet in blue. LIR.Low (*n* = 28). LIR.High (*n* = 19). MIR. Low (*n* = 34). MIR. High (*n* = 36).
**FIgure S7:** Estimated marginal means for HOMA‐IR by IR metabotype and MH tertiles across diets. A 2 × 2 matrix shows estimated total fat percentage grouped by IR metabotype (MIR vs. LIR) and MH tertiles (high vs. low). The low‐fat. high‐protein high‐fiber (LFHP) diet is in yellow and the high‐monounsaturated fat (HMUFA) diet in blue. LIR.Low (*n* = 28). LIR.High (*n* = 19). MIR. Low (*n* = 34). MIR. High (*n* = 36).
**FIgure S8:** Estimated marginal means for HOMA‐B by IR metabotype and MH tertiles across diets. A 2 × 2 matrix shows estimated total fat percentage grouped by IR metabotype (MIR vs. LIR) and MH tertiles (high vs. low). The low‐fat. high‐protein high‐fiber (LFHP) diet is in yellow and the high‐monounsaturated fat (HMUFA) diet in blue. LIR.Low (*n* = 28). LIR.High (*n* = 19). MIR. Low (*n* = 34). MIR.High (*n* = 36).
**FIgure S9:** Estimated marginal means for Matsuda index by IR metabotype and MH tertiles across diets. A 2 × 2 matrix shows estimated total fat percentage grouped by IR metabotype (MIR vs. LIR) and MH tertiles (high vs. low). The low‐fat. high‐protein high‐fiber (LFHP) diet is in yellow and the high‐monounsaturated fat (HMUFA) diet in blue. LIR.Low (*n* = 28). LIR.High (*n* = 19). MIR.Low (*n* = 34). MIR.High (*n* = 36).
**FIgure S10:** Estimated marginal means for disposition index by IR metabotype and MH tertiles across diets. A 2 × 2 matrix shows estimated total fat percentage grouped by IR metabotype (MIR vs. LIR) and MH tertiles (high vs. low). The low‐fat. high‐protein high‐fiber (LFHP) diet is in yellow and the high‐monounsaturated fat (HMUFA) diet in blue. LIR.Low (*n* = 28). LIR.High (*n* = 19). MIR.Low (*n* = 34). MIR.High (*n* = 36).
**FIgure S11:** Estimated marginal means for CRP by IR metabotype and MH tertiles across diets. A 2 × 2 matrix shows estimated total fat percentage grouped by IR metabotype (MIR vs. LIR) and MH tertiles (high vs. low). The low‐fat. high‐protein high‐fiber (LFHP) diet is in yellow and the high‐monounsaturated fat (HMUFA) diet in blue. LIR.Low (*n* = 28). LIR.High (*n* = 19). MIR.Low (*n* = 34). MIR.High (*n* = 36).
**FIgure S12:** Estimated marginal means for triacylglycerides by IR metabotype and MH tertiles across diets. A 2 × 2 matrix shows estimated total fat percentage grouped by IR metabotype (MIR vs. LIR) and MH tertiles (high vs. low). The low‐fat. high‐protein high‐fiber (LFHP) diet is in yellow and the high‐monounsaturated fat (HMUFA) diet in blue. LIR.Low (*n* = 28). LIR.High (*n* = 19). MIR.Low (*n* = 34). MIR.High (*n* = 36).

**Table S1:** Tested covariates and determinants of DXA‐derived body composition and metabolic outcomes.
**Table S2:** Mixed linear regression model associating DXA body composition with the interaction effect of metabotypes and high versus low MetaboHealth tertile across the interaction (diet and time) adjusted for covariates age and sex. Nominally significant values (*p* < 0.05) are highlighted in yellow.
**Table S3:** Mixed linear regression model associating metabolic health outcomes with the interaction effect of metabotypes and high versus low MetaboHealth tertile across the interaction (diet and time) adjusted for covariates age, sex, and BMI. Nominally significant values (*p* < 0.05) are highlighted in yellow.
**Table S4:** Estimated marginal means associating DXA body composition with the interaction effect of metabotypes and high versus low MetaboHealth tertile across the interaction (diet and time point) adjusted for covariates.
**Table S5:** Estimated marginal means associating cardiometabolic health outcomes with the interaction effect of metabotypes and high versus low MetaboHealth tertile across the interaction (diet and time point) adjusted for covariates.

## Data Availability

Data described in the manuscript, code book, and analytic code will be made available upon request pending application and approval to coauthors.
