# Supplementary material for: The MetaboHealth Score Enhances Insulin Resistance Metabotyping for Targeted Fat Loss: The PERSON Study
Source: Obesity (Silver Spring). 2026 Jan 14;34(3):550–64. doi: 10.1002/oby.70116 (PMC12933231; doi:10.1002/oby.70116)

**FIGURE S1. FIGURE S4. Estimated marginal means for lean mass index by IR metabotype and MH tertiles across diets.** A 2x2 matrix shows estimated total fat percentage grouped by IR metabotype (MIR vs. LIR) and MH tertiles (high vs. low). The low-fat. high-protein. high-fiber (LFHP) diet is in yellow and the high-monounsaturated fat (HMUFA) diet in blue. LIR.Low (n=28). LIR.High (n=19). MIR.Low (n=34). MIR.High (n=36).


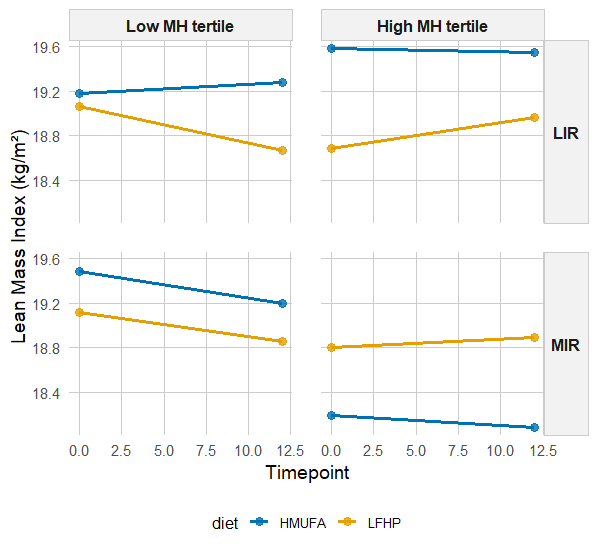


**FIGURE S2. Estimated marginal means for appendicular lean mass index by IR metabotype and MH tertiles across diets.** A 2x2 matrix shows estimated total fat percentage grouped by IR metabotype (MIR vs. LIR) and MH tertiles (high vs. low). The low-fat. high-protein. high-fiber (LFHP) diet is in yellow and the high-monounsaturated fat (HMUFA) diet in blue. LIR.Low (n=28). LIR.High (n=19). MIR.Low (n=34). MIR.High (n=36).


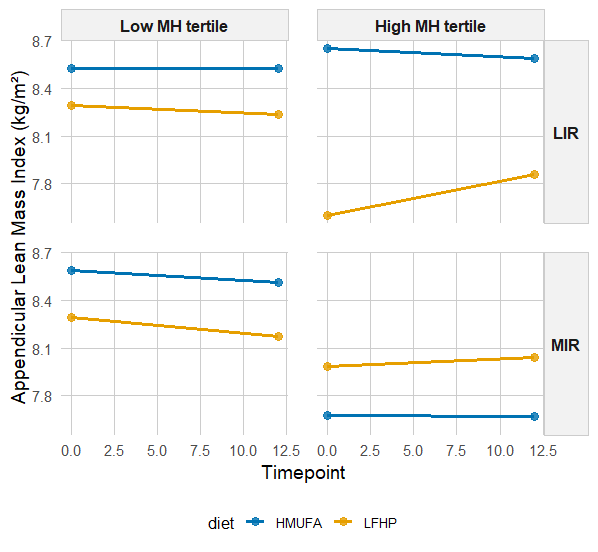


**FIGURE S3. Estimated marginal means for VAT by IR metabotype and MH tertiles across diets.** A 2x2 matrix shows estimated total fat percentage grouped by IR metabotype (MIR vs. LIR) and MH tertiles (high vs. low). The low-fat. high-protein. high-fiber (LFHP) diet is in yellow and the high-monounsaturated fat (HMUFA) diet in blue. LIR.Low (n=28). LIR.High (n=19). MIR.Low (n=34). MIR.High (n=36).


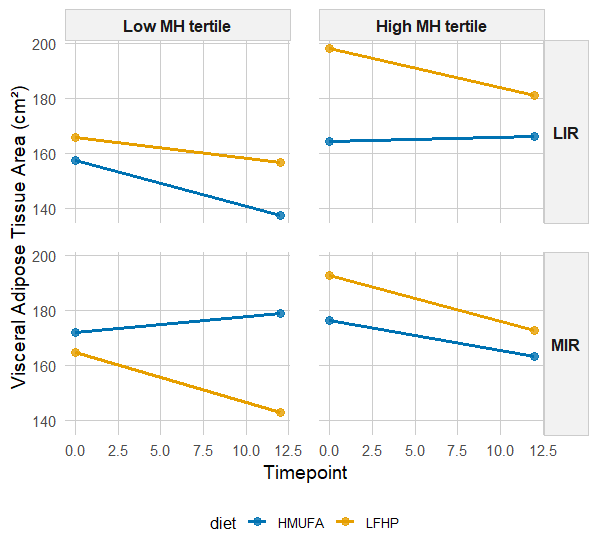


**FIGURE S4. Estimated marginal means for MetaboHealth score by IR metabotype and MH tertiles across diets.** A 2x2 matrix shows estimated total fat percentage grouped by IR metabotype (MIR vs. LIR) and MH tertiles (high vs. low). The low-fat. high-protein high-fiber (LFHP) diet is in yellow and the high-monounsaturated fat (HMUFA) diet in blue. LIR.Low (n=28). LIR.High (n=19). MIR.Low (n=34). MIR.High (n=36).


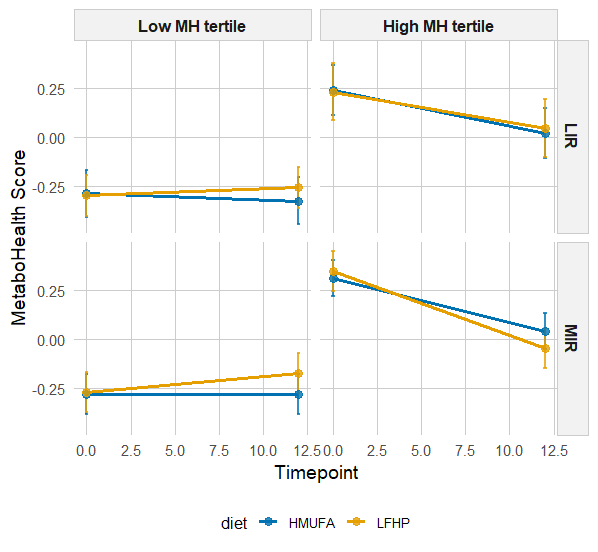


**FIGURE S5. Estimated marginal means for MISI by IR metabotype and MH tertiles across diets.** A 2x2 matrix shows estimated total fat percentage grouped by IR metabotype (MIR vs. LIR) and MH tertiles (high vs. low). The low-fat. high-protein high-fiber (LFHP) diet is in yellow and the high-monounsaturated fat (HMUFA) diet in blue. LIR.Low (n=28). LIR.High (n=19). MIR.Low (n=34). MIR.High (n=36).


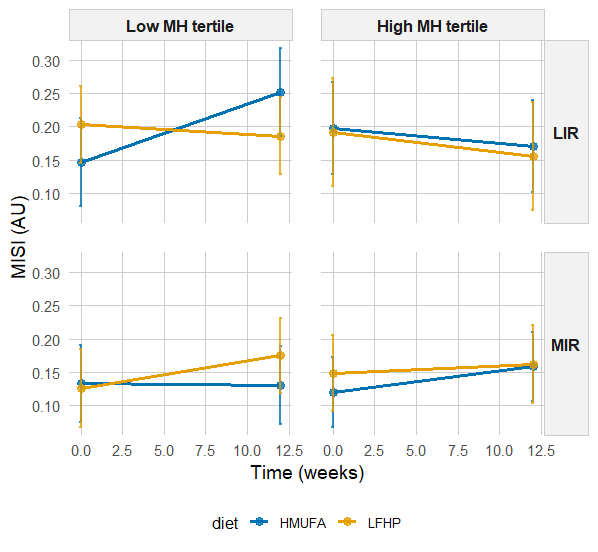


**FIGURE S6. Estimated marginal means for HIRI by IR metabotype and MH tertiles across diets.** A 2x2 matrix shows estimated total fat percentage grouped by IR metabotype (MIR vs. LIR) and MH tertiles (high vs. low). The low-fat. high-protein high-fiber (LFHP) diet is in yellow and the high-monounsaturated fat (HMUFA) diet in blue. LIR.Low (n=28). LIR.High (n=19). MIR.Low (n=34). MIR.High (n=36).


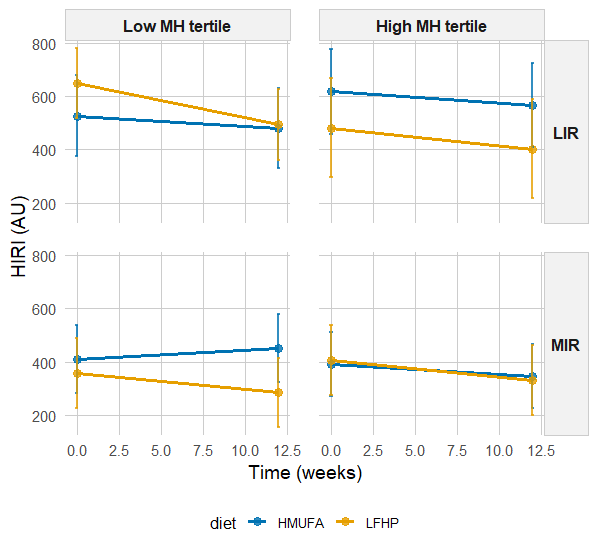


**FIGURE S7. Estimated marginal means for HOMA-IR by IR metabotype and MH tertiles across diets.** A 2x2 matrix shows estimated total fat percentage grouped by IR metabotype (MIR vs. LIR) and MH tertiles (high vs. low). The low-fat. high-protein high-fiber (LFHP) diet is in yellow and the high-monounsaturated fat (HMUFA) diet in blue. LIR.Low (n=28). LIR.High (n=19). MIR.Low (n=34). MIR.High (n=36).


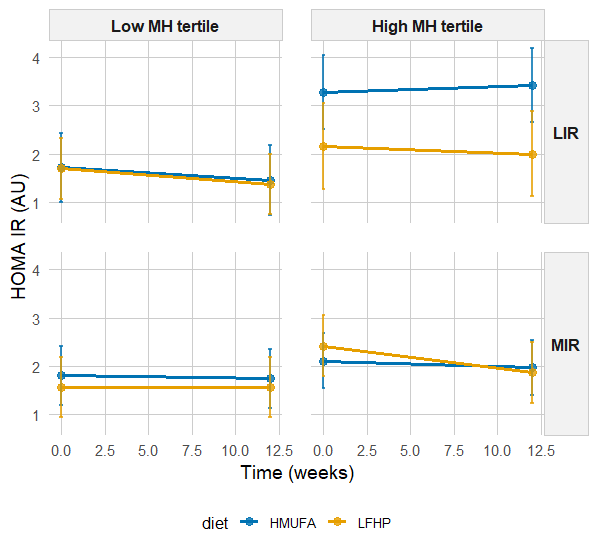


**FIGURE S8. Estimated marginal means for HOMA-B by IR metabotype and MH tertiles across diets.** A 2x2 matrix shows estimated total fat percentage grouped by IR metabotype (MIR vs. LIR) and MH tertiles (high vs. low). The low-fat. high-protein high-fiber (LFHP) diet is in yellow and the high-monounsaturated fat (HMUFA) diet in blue. LIR.Low (n=28). LIR.High (n=19). MIR.Low (n=34). MIR.High (n=36).


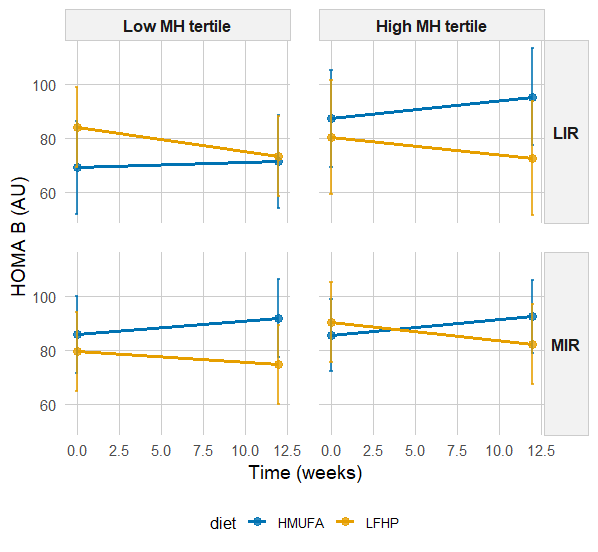


**FIGURE S9. Estimated marginal means for Matsuda Index by IR metabotype and MH tertiles across diets.** A 2x2 matrix shows estimated total fat percentage grouped by IR metabotype (MIR vs. LIR) and MH tertiles (high vs. low). The low-fat. high-protein high-fiber (LFHP) diet is in yellow and the high-monounsaturated fat (HMUFA) diet in blue. LIR.Low (n=28). LIR.High (n=19). MIR.Low (n=34). MIR.High (n=36).


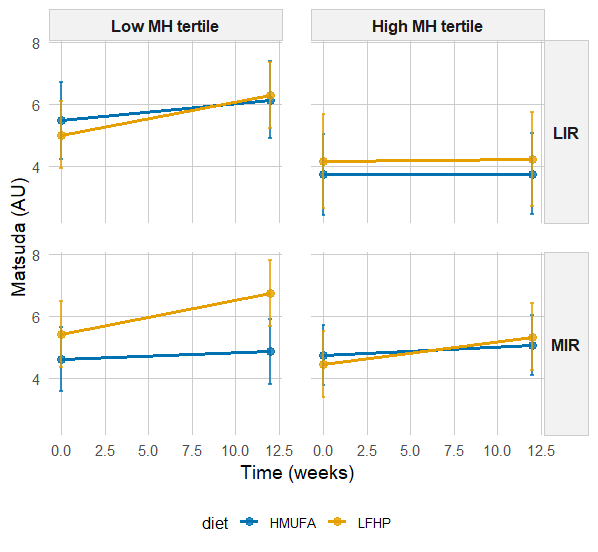


**FIGURE S10. Estimated marginal means for Disposition Index by IR metabotype and MH tertiles across diets.** A 2x2 matrix shows estimated total fat percentage grouped by IR metabotype (MIR vs. LIR) and MH tertiles (high vs. low). The low-fat. high-protein high-fiber (LFHP) diet is in yellow and the high-monounsaturated fat (HMUFA) diet in blue. LIR.Low (n=28). LIR.High (n=19). MIR.Low (n=34). MIR.High (n=36).


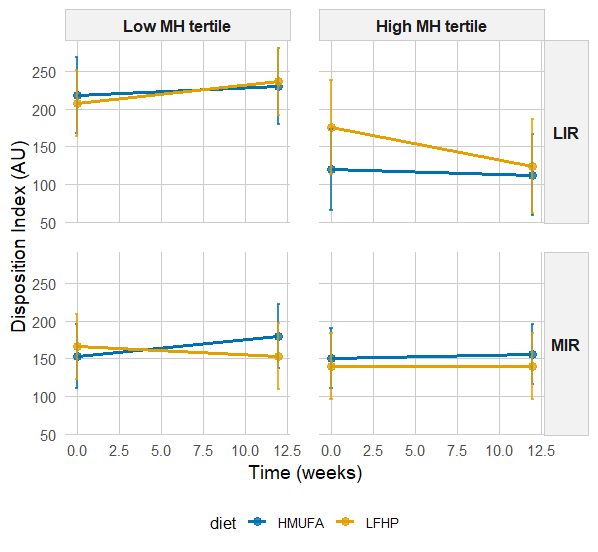


**FIGURE S11. Estimated marginal means for CRP by IR metabotype and MH tertiles across diets.** A 2x2 matrix shows estimated total fat percentage grouped by IR metabotype (MIR vs. LIR) and MH tertiles (high vs. low). The low-fat. high-protein high-fiber (LFHP) diet is in yellow and the high-monounsaturated fat (HMUFA) diet in blue. LIR.Low (n=28). LIR.High (n=19). MIR.Low (n=34). MIR.High (n=36).


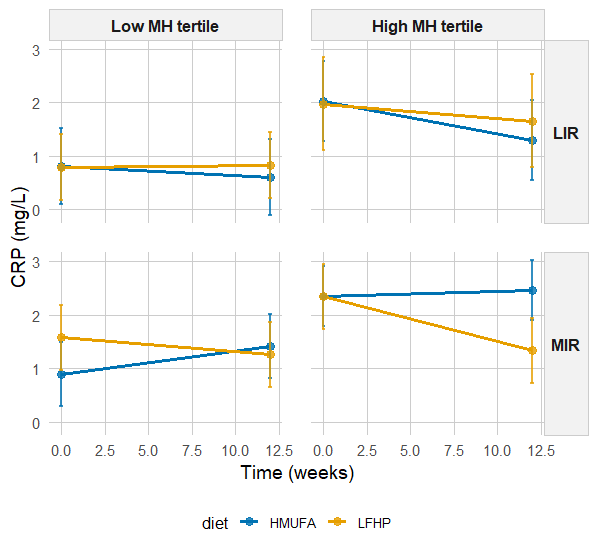


**FIGURE S12. Estimated marginal means for TAG by IR metabotype and MH tertiles across diets.** A 2x2 matrix shows estimated total fat percentage grouped by IR metabotype (MIR vs. LIR) and MH tertiles (high vs. low). The low-fat. high-protein high-fiber (LFHP) diet is in yellow and the high-monounsaturated fat (HMUFA) diet in blue. LIR.Low (n=28). LIR.High (n=19). MIR.Low (n=34). MIR.High (n=36).


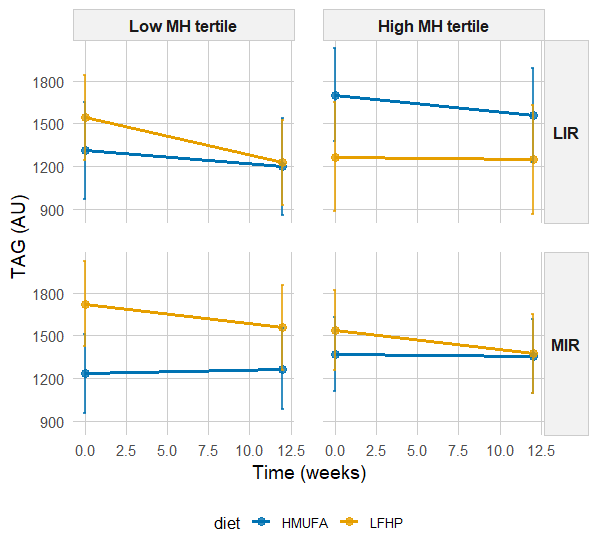

Supplement: Supplementary file 1 — Figure S1: Estimated marginal means for lean mass index by IR metabotype and MH tertiles across diets. A 2 × 2 matrix shows estimated total fat percentage grouped by IR metabotype (MIR vs. LIR) and MH tertiles (high vs. low). The low‐fat. high‐protein. high‐fiber (LFHP) diet is in yellow and the high‐monounsaturated fat (HMUFA) diet in blue. LIR.Low (n = 28). LIR.High (n = 19). MIR.Low (n = 34). MIR.High (n = 36). FIgure S2: Estimated marginal means for appendicular lean mass index by IR metabotype and MH tertiles across diets. A 2 × 2 matrix shows estimated total fat percentage grouped by IR metabotype (MIR vs. LIR) and MH tertiles (high vs. low). The low‐fat. high‐protein. high‐fiber (LFHP) diet is in yellow and the high‐monounsaturated fat (HMUFA) diet in blue. LIR.Low (n = 28). LIR.High (n = 19). MIR.Low (n = 34). MIR.High (n = 36). FIgure S3: Estimated marginal means for VAT by IR metabotype and MH tertiles across diets. A 2 × 2 matrix shows estimated total fat percentage grouped by IR metabotype (MIR vs. LIR) and MH tertiles (high vs. low). The low‐fat. high‐protein. high‐fiber (LFHP) diet is in yellow and the high‐monounsaturated fat (HMUFA) diet in blue. LIR.Low (n = 28). LIR.High (n = 19). MIR.Low (n = 34). MIR.High (n = 36). FIgure S4: Estimated marginal means for MetaboHealth score by IR metabotype and MH tertiles across diets. A 2 × 2 matrix shows estimated total fat percentage grouped by IR metabotype (MIR vs. LIR) and MH tertiles (high vs. low). The low‐fat. high‐protein high‐fiber (LFHP) diet is in yellow and the high‐monounsaturated fat (HMUFA) diet in blue. LIR.Low (n = 28). LIR.High (n = 19). MIR.Low (n = 34). MIR.High (n = 36). FIgure S5: Estimated marginal means for MISI by IR metabotype and MH tertiles across diets. A 2 × 2 matrix shows estimated total fat percentage grouped by IR metabotype (MIR vs. LIR) and MH tertiles (high vs. low). The low‐fat. high‐protein high‐fiber (LFHP) diet is in yellow and the high‐monounsaturated fat (HMUFA) diet in [file OBY-34-550-s002.docx]
